# Design and evaluation of novel triazole derivatives as potential anti-gout inhibitors: a comprehensive molecular modeling study

**DOI:** 10.3389/fchem.2025.1518777

**Published:** 2025-03-06

**Authors:** Mohammed Er-rajy, Mohamed El fadili, Sara Zarougui, Somdutt Mujwar, Mourad Aloui, Mohammed Zerrouk, Belkheir Hammouti, Larbi Rhazi, Rachid Sabbahi, Mohammed M. Alanazi, Khalil Azzaoui, Rachid Salghi, Menana Elhallaoui

**Affiliations:** ^1^ LIMAS Laboratory, Faculty of Sciences Dhar El Mahraz, Sidi Mohamed Ben Abdellah University, Fez, Morocco; ^2^ Euromed University of Fes, UMF, Fez, Morocco; ^3^ Chitkara College of Pharmacy, Chitkara University, Rajpura, Punjab, India; ^4^ Engineering Laboratory of Organometallic, Molecular Materials and Environment, Faculty of Sciences, Sidi Mohamed Ben Abdellah University, Fez, Morocco; ^5^ Institut Polytechnique UniLaSalle, Université d’Artois, Beauvais, France; ^6^ Research Team in Science and Technology, Higher School of Technology, Ibn Zohr University, Laayoune, Morocco; ^7^ Department of Pharmaceutical Chemistry, College of Pharmacy, King Saud University, Riyadh, Saudi Arabia; ^8^ Laboratory of Industrial Engineering, Energy and the Environment (LI3E) SUPMTI, Rabat, Morocco; ^9^ Laboratory of Applied Chemistry and Environment, National School of Applied Sciences, University Ibn Zohr, Agadir, Morocco

**Keywords:** 3D-QSAR, anti-gout drugs, molecular docking, molecular dynamic, conception of novel derivatives, ADMET proprieties, DFT study

## Abstract

**Introduction:**

Gout is the most common inflammatory arthritis, characterized by hyperuricemia, tophus formation, joint disease, and kidney stones. Uric acid, the final byproduct of purine catabolism, is eliminated via the kidneys and digestive system. Xanthine oxidase (XO) catalyzes the conversion of hypoxanthine and xanthine into uric acid, making XO inhibitors crucial for treating hyperuricemia and gout. Currently, three XO inhibitors are clinically used, showing significant efficacy. A molecular modeling study on triazole derivatives aims to identify novel XO inhibitors using 3D-QSAR, molecular docking, MD simulations, ADMET analysis, and DFT calculations. These computational approaches facilitate drug discovery while reducing research costs.

**Methods:**

Our work focuses on a series of synthesized anti-xanthine oxidase inhibitors, aiming to develop new inhibitors. A computational study was carried out to identify the xanthine oxidase inhibitory structural features of a series of triazole inhibitors using computational method.

**Results:**

A model based on CoMFA and CoMSIA/SEA has been built to predict new triazole derivatives.

**Discussion:**

The optimal model established from CoMFA and CoMSIA/SEA was successfully evaluated for its predictive capability. Visualization of the contour maps of both models showed that modifying the substituents plays a key role in enhancing the biological activity of anti-gout inhibitors. Molecular docking results for complexes N°8-3NVY and N°22-3NVY showed scores of −7.22 kcal/mol and −8.36 kcal/mol, respectively, indicating substantial affinity for the enzyme. Complex N°8-3NVY forms two hydrogen bonds with SER 69 and ASN 71, three alkyl bonds with ALA 70, LEU 74, and ALA 75, and one Pi-Pi T-shaped bond with PHE 68. Complex N°22-3NVY forms three hydrogen bonds with HIS 99, ARG 29, and ILE 91, and one halogen bond with LEU 128 at 3.60 Å. A MD study revealed that the N°22-3NVY complex remained highly stable throughout the simulation. Therefore, we proposed six new molecules, their anti-gout inhibitory activities were predicted using two models, and they were evaluated for Lipinski's rule, and ADMET properties. The results show that both Pred 4 and Pred 5 have better pharmacokinetic properties than the height potent molecule in the studied series, making these two compounds valuable candidates for new anti-gout drugs. Subsequently, using DFT study to evaluate the chemical reactivity properties of these two proposed compounds, the energy gap results revealed that both molecules exhibit moderate chemical stability and reactivity.

## 1 Introduction

Globally, gout is the most frequent cause of inflammatory arthritis (Dalbeth et al., 2021). It is a chronic condition marked by high serum uric acid levels and is characterized by hyperuricemia, tophus formation, joint disease, and kidney stones (Ragab et al., 2017). The last byproduct of purine catabolism in humans is uric acid, which is eliminated via the kidneys and digestive system. In the purine metabolism pathway, xanthine oxidase (XO) is an essential and rate-limiting enzyme that catalyzes the conversion of xanthine and hypoxanthine into uric acid (Furuhashi, 2020). Thus, in the therapy of hyperuricemia and gout, XO inhibitors can help individuals who either overproduce or under excrete uric acid. In fact, XO inhibitors—like allopurinol—are frequently prescribed to gout patients as the initial course of urate-lowering treatment (Linani et al., 2022).

According to the worldwide epidemiology of gout, the disease’s prevalence ranges from 0·3%–3% in Europe (0·9% in metropolitan France), 7 3.9% in the United States of America (with US Asian people reaching 6.6%), 8, 9 and 4·9% in Taiwan (Pascart et al., 2024).

Currently, three xanthine oxidase inhibitors (allopurinol, febuxostat, and topiroxostat) are clinically used to treat hyperuricemia and gout, demonstrating significant therapeutic effects in clinical practice (Tang et al., 2018). Allopurinol, a purine analog approved in 1966, has served as an anti-gout medication for more than 50 years. To this day, it remains the most widely prescribed treatment for hyperuricemia and gout around the globe (Sato et al., 2018). This highlights the urgent need for the development of novel drug compounds with enhanced efficacy and specificity to combat the disease and address emerging challenges in treatment. However, the synthesis of a series of 4-(phenoxymethyl)-1H-1,2,3-triazole is based on marketed drugs such as allopurinol, febuxostat and topiroxostat, which have been applied clinically for the treatment of gout and have achieved clear curative effects in clinical practice (Cicero et al., 2021).

A series of synthesized 4-(phenoxymethyl)-1H-1,2,3-triazole compounds were evaluated biologically by Zhang et al. (2022). This series is the subject of a molecular modeling study aimed at proposing other candidate XO enzyme inhibitors. The quantitative structure-activity relationship (3D-QSAR) studies, molecular docking, Molecular dynamic (MD) simulation and the absorption, distribution, metabolism, excretion, and toxicity (ADMET) properties studies are crucial modeling methods that generate predictive and robust models for identifying new drug candidates, thereby reducing economic investment costs (Er-rajy et al., 2022a; Jitonnom et al., 2024; Zarougui et al., 2024).

The 3D-QSAR methodology allows for the establishment of a quantitative correlation between dependent variations, such as pIC_50_ (µM) biological activity, and molecular structures or properties to create a valid mathematical model. The Comparative Molecular Field Analysis (CoMFA) and Comparative Molecular Similarity Indices Analysis (CoMSIA) is the most commonly used method to build 3D-QSAR models. To assess the predictive capability and robustness of the models, such as CoMFA and CoMSIA/SEA, external and internal validation were conducted to ensure the absence of outliers in the data set (Tsai et al., 2010; Er-rajy et al., 2023b; Er-Rajy et al., 2024a). In addition, molecular docking studies were carried out to predict the ligands’ optimized binding conformation and to understand the differences in structural interactions between the most active ligands and their target protein, followed by a molecular dynamics study of docked compounds to assess their stability during 100 ns of simulation (Bouammali et al., 2023; Faris et al., 2023).

Based on the results of our work, we proposed new compounds as potential inhibitors of the OX enzyme. Finally, we evaluated the drug-like properties of these new compounds by testing their pharmacokinetic parameters (Ferreira and Andricopulo, 2019). Afterwards, we carried out a reactivity study of the molecules we proposed, using density functional theory (DFT) with the 6-311G (d, p) basis set and the Becke, 3-parameter, Lee-Yang-Parr (B3LYP) functional (Ziegler, 1991; Zarougui et al., 2023). Next, we constructed Non-Covalent Interaction analysis (NCI) diagrams, which provide valuable insights into the interactions between atoms in doping, often referred to as weak forces. Subsequently, a Reduced Density Gradient (RDG) study was conducted to analyze and visualize molecular interactions within a chemical system (Amraoui et al., 2024; Bouayad et al., 2024). This tool highlights regions of non-covalent interactions, such as hydrogen bonds, van der Waals forces, and repulsive interactions, providing a clear and intuitive visual representation of their nature and strength.

## 2 Materiel and methods

### 2.1 The study database

We based for the construction of the different models CoMFA, and CoMSIA, on an experimental data set (26 derivatives) synthesized by Zhang et al. (2022); Table 1), and evaluated by their XO inhibitory activities [IC_50_ (µM)]. The training set and the test set are the two sets into which we separated the experimental data set (Er-rajy et al., 2023c).

**TABLE 1:** The 4-(phenoxymethyl)-1H-1,2,3-triazole derivatives as novel XO inhibitors: biological capacities and structures.

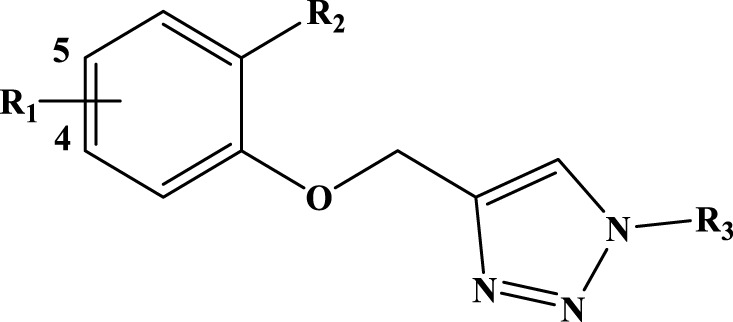					
N°	R_1_	R_2_	R_3_	IC_50_	pIC_50_
1	H	CHO	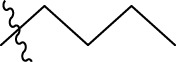	13.5	4.870
2	H	CHO	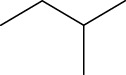	21.14	4.675
3	H	CHO	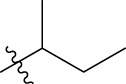	21.78	4.662
4	H	CHO	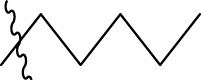	18.14	4.741
5	H	CHO	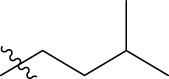	16.03	4.795
6^a^	H	CHO	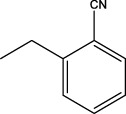	2.22	5.654
7	H	CHO	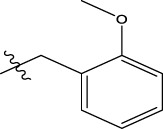	1.45	5.839
8	H	CHO	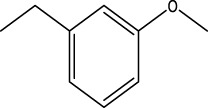	1.02	5.991
9	H	CHO	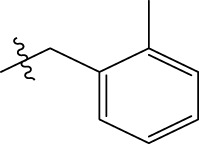	1.63	5.788
10	H	CHO	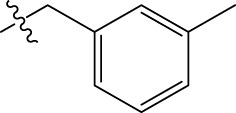	1.11	5.955
11^a^	H	5-F	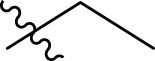	3.46	5.461
12	H	5-F	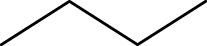	5.24	5.281
13	H	5-F	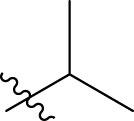	2.56	5.592
14	H	5-F	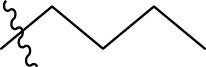	2.49	5.604
15	5-F	CHO	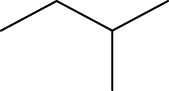	14.32	4.844
16	5-F	CHO	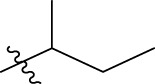	9.36	5.029
17	5-F	CHO	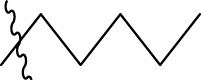	2.99	5.524
18	5-F	CHO	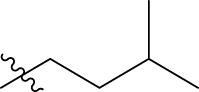	2.12	5.674
19^a^	5-F	CHO	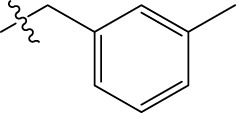	21.51	4.667
20	5-F	CHO	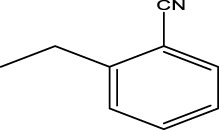	4.15	5.382
21	5-F	CHO	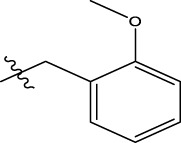	0.94	6.027
22	5-F	CHO	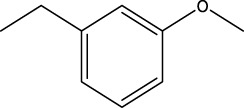	0.7	6.155
23^a^	5-F	CHO	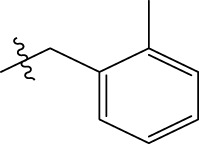	4.01	5.397
24^a^	5-F	CHO	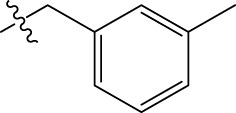	1.18	5.928
25	H	CHO	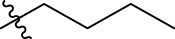	3.59	5.445
26	4-F	CHO	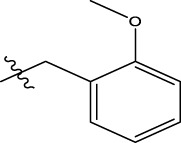	2.07	5.684

^a^ Test set, R: radical, N°: number.

### 2.2 Model construction and performance analysis

SYBYL-X 2.1 was used to minimization, alignment derivatives and to constructed CoMFA and CoMSIA fields (Bringmann and Rummey, 2003). For every network, CoMFA descriptors were constructed with a grid spacing of 1 unit, ranging up to 4 units in all three dimensions within the specified region (Böhm et al., 1999). We determined the energies of the steric and electrostatic fields with Van der Waals potential, Coulombic terms, and a sp^3^ hybridized carbon atom with a +1e charge as the probe atom using the standard Tripos force field, values have been truncated at 30 kcal/mol (Er-rajy et al., 2023a), For CoMSIA, a distance-dependent Gaussian type of physicochemical characteristic was chosen to avoid singularities at atomic positions. We used similar standard parameters for steric (S), electrostatic (E), acceptor hydrogen (A), donor hydrogen (D) and hydrophobic (H) effects in the CoMSIA field calculation, without arbitrary constraints specific to the CoMFA field calculation (Guo et al., 2005; Baidya et al., 2023).

The characteristics of the anti-gout inhibitors, which are connected to their activity, were evaluated using the Partial Least Squares (PLS) approach. We assessed the models’ relevance using the cross-validation correlation coefficient (Q^2^), the coefficient of determination (R^2^), the optimal number of principal components (NOC) and the standard error of estimate (SEE). Subsequently, various CoMFA and CoMSIA models were developed through non-validated PLS analysis (Aloui et al., 2024; Er-Rajy et al., 2024b).

The robustness of the 3D-QSAR model increases as Q^2^ > 0.5 and 1 < NOC <6. After selecting the optimal values for NOC and Q2 for the 3D-QSAR modeling, we assess the overall significance of the developed model by calculating statistical parameters such as the coefficient R^2^, SEE error. A 3D-QSAR model is considered reliable if R^2^ > 0.6, and SEE is low, as it will then provide an accurate description of pIC_50_ values based on the structural properties of the investigated molecules (Roy and Pratim Roy, 2009; Nour et al., 2022a; Er-rajy et al., 2023d).

### 2.3 Molecular docking study

Before the docking study, the two most active molecule drawn in ChemDraw 16.0, and then the geometry was MM2 optimized (Allinger, 1977). This step ensured the proper equilibrium of the system and allowed for the verification of the protonation states and polar hydrogen atoms of the ligands in an aqueous environment. On the other hand, the first step involved preparing the backbone structure of the protein and the ligands (Er-rajy et al., 2025). The protein preparation included removing water molecules and non-protein elements, adding polar hydrogen atoms, and assigning Gasteiger charges (Nour et al., 2022b).

Both compounds were tested for their anti-gout activity. For the antigout study, we utilized the xanthine oxidase receptor extracted from the Protein Data Bank (PDB ID: 3NVY with a resolution of 2.00 Å) (Cao et al., 2014). A grid was created with parameters X = 39.948, Y = −17.942 and Z = 24.367 Å.

We used AutoDockTools software to carry out the molecular docking (Holt et al., 2008). Following Lamarck’s genetic algorithm (LGA), docking studies of the protein-ligand complex were conducted to obtain the lowest binding free energy (∆G). For molecular docking studies, we used a grid of 40*40*40 points in the X, Y and Z directions. In the molecular docking studies, we used a total number of 100 solutions calculated in each case, using a population size of 350.

After preparing the molecules under study and the complexes, including the removal of water molecules, we carry out the molecular docking protocol (Hjouji et al., 2025). Finally, to observe the ligand-protein interaction and eliminate water molecules, curation of missing side-chain residues, and merge non-polar hydrogens, we used Discovery 2021 software (Pinzi and Rastelli, 2019).

### 2.4 Molecular dynamics

The two ligands docked were chosen for MD simulations based on the molecular docking results in order to ascertain the stability of the molecular bonds with the target protein (Parihar et al., 2022). Using the Desmond program and Maestro of the Schrödinger Suite, the OPLS3e force field, MD was run for 100 ns (Miao et al., 2019). By solving it in a 10-size aqueous simulation box, the OPLS3e force field was utilized for the MD simulation of two complexes and ligands bound (the most active) to the base receptor. For a 10 Å region, explicit water molecules were modeled using the TIP3P water model under orthorhombic periodic boundary conditions (Bizzarri and Cannistraro, 2002). Counterions such as Na⁺ and Cl⁻ were added to the system to neutralize it, and their positions were energy-minimized. Using the Particle Mesh Ewald (PME) approach, long-range electrostatic connections between the protein and the complexed ligands were calculated for Coulombic interactions, after the MD simulation of the NPT ensemble of Nose-Hoover thermostat was run (Petersen, 1995; Jang et al., 2002).

In our MD simulations, a cutoff radius of 9.0 Å was used for Coulombic interactions, with a grid spacing of 0.8 Å for the PME method. Simulations were carried out under NPT ensemble conditions, using the Nose-Hoover thermostat and barostat to maintain constant temperature (300 K) and pressure (1 bar). The equations of motion were integrated using Velocity Verlet with a time step of 2 fs? The interaction diagram module of the Desmond simulation package was utilized to determine the precise binding interactions between the ligand and the viral protein.

### 2.5 ADMET proprieties

A comprehensive grasp of ADMET properties is indispensable in evaluating the feasibility of potential therapeutics within the swiftly evolving realm of drug discovery (Sinha and Vohora, 2018). Just as vital is a compound’s “drug-likeness,” indicating its potential for successful development as an orally administered medication. To this end, our study used the web servers SwissADME and pkCSM to carry out in-depth *in silico* analysis of the molecules N°22 and N°8, as well as for the proposed new compounds (Pires et al., 2015; Daina et al., 2017).

We have taken into account factors such as lipophilicity, water solubility, saturation, flexibility, and various pharmacokinetic properties, all of which are crucial to a drug’s bioavailability. Furthermore, drug similarity assessment was conducted based on established pharmaceutical guidelines, including Lipinski’s five Rules (Lipinski, 2004; Kowalska et al., 2018).

### 2.6 DFT study

In quantum mechanics, DFT study has established itself as the leading method for exploring the electronic and chemical spectroscopic characteristics of molecules in the field of quantum mechanics (Orio et al., 2009). This report, focusing on quantum mechanical principles, showcases molecular structures visualized using Gauss View 6.0.16. Theoretical evaluations of all computational models were conducted utilizing Gaussian 09 software (Tomberg, 2013).

Initial DFT calculations were performed on the two newly predicted molecules. Geometrical parameters for all configurations were determined using the DFT method with the 6-311G (d, p) basis set and B3LYP functional. This choice was based on its established reliability in previous literature concerning these molecules (Ayub et al., 2024; Edder et al., 2024). This configuration seemed optimal for assessing the reactive properties of the molecules. To better understand the relationships between the various components of the compound, topological analyses were conducted using VMD and the Multiwfn program, this approach relies on non-covalent interaction density calculations to enhance the detection of interactions within the studied structures (Salah et al., 2023; Spivak et al., 2023).

## 3 Results and discussion

### 3.1 Alignments and analysis of 3D-QSAR models

**FIGURE 1:**
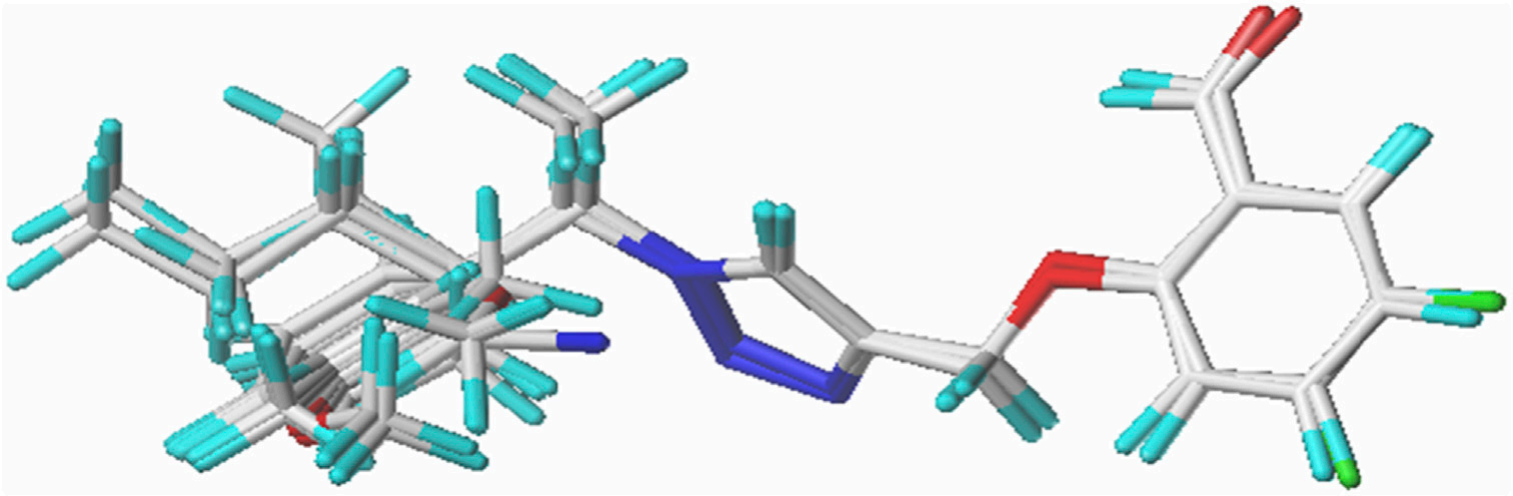
Structure superposition of database.

To construct a robust and dependable model, all the compounds must be superimposed with great precision. All derivatives of the molecules were added to a database and aligned to the common core using the Sybyl X-2.1 rigid docking method, with compound N°22 (the most active) serving as the template (Figure 1.).

After several attempts, we constructed two QSAR models. Then, we calculated the predicted activities for each model as well as the residuals for each molecule. Table 2 summarizes the residuals from the various models (CoMFA and CoMSIA/SEA), as well as the observed and predicted activity of each molecule.

**TABLE 2:** The examined compounds’ residuals and predicted pIC_50_.

N° of molecules	pIC_50_ observed	pIC_50_ predict			
		CoMFA	Residues	CoMSIA/SEA	Residues
1	4.870	4.8071	4.8071	4.7569	0.1131
2	4.675	4.628	4.6280	4.6728	0.0022
3	4.662	4.5712	4.5712	4.6046	0.0574
4	4.741	4.9405	4.9405	4.8744	−0.1334
5	4.795	5.1809	5.1809	5.1255	−0.3305
6^a^	5.654	5.139	5.1390	5.2981	0.3559
7	5.839	5.7491	5.7491	5.7096	0.1294
8	5.991	5.9800	5.980	5.958	0.033
9	5.788	5.6251	5.6251	5.6454	0.1426
10	5.955	5.7694	5.7694	5.7595	0.1955
11^a^	5.461	5.2957	5.2957	5.3384	0.1226
12	5.281	5.293	5.2930	5.3157	−0.0347
13	5.592	5.2434	5.2434	5.2645	0.3275
14	5.604	5.4921	5.4921	5.4656	0.1384
15	4.844	5.092	5.0920	5.2289	−0.3849
16	5.029	5.3404	5.3404	5.3591	−0.3301
17	5.524	5.492	5.4920	5.4631	0.0609
18	5.674	5.4877	5.4877	5.4744	0.1996
19^a^	4.667	5.8019	5.8019	4.5997	0.0673
20	5.382	5.4336	5.4336	5.4516	−0.0696
21	6.027	6.0574	6.0574	6.0602	−0.0332
22	6.155	6.3037	6.3037	6.3166	−0.1616
23^a^	5.397	5.9297	5.9297	5.6606	−0.2636
24^a^	5.928	6.0546	6.0546	5.7235	0.2045
25	5.445	5.2501	5.2501	5.2263	0.2187
26	5.684	5.8185	5.8185	5.8224	−0.1384

^a^ Test set.

QSAR models have been proposed to explain and quantitatively predict the anti-gout inhibitory activities of a series of 4-(phenoxymethyl)-1H-1,2,3-triazoles. The residual values presented in Table 2 are close to zero for most molecules and reach a maximum of about 0.3 for some, which means that the two models we have proposed are very robust.

### 3.2 Validation of the model built

The independent variables gathered from the training set were subjected to PLS cross-validation analysis in order to ascertain the proper statistical parameters. The outcomes for the various statistical models and the corresponding statistical parameters are presented in Table 3.

**TABLE 3:** Model statistics based on the PLS approach for the two models CoMFA and CoMSIA.

	$Q^{2}$	$R^{2}$	Error	NOC	$R_{\text{ext}\;}^{2}$	Fraction		
						Steric	Electrostatic	Acceptor
CoMFA	0.652	0.859	0.195	2	0.683	0.684	0.316	
CoMSIA SEA	0.676	0.805	0.229	2	0.767	0.253	0.665	0.082

Q^2^: The Cross-validated correlation coefficient, R^2^: The non-validated correlation coefficient, NOC: the optimal number of principal components, SEE: Standard estimation of error.

As illustrated in Table 3, for the CoMFA model accounts for 68.4% and 31.6% of the variance in the steric and electrostatic fields, respectively. The training set’s coefficient $Q_{\text{cv}}^{2}$ and R^2^ have values of 0.652 and 0.859, respectively, and the ideal NOC to use is two, and also a lower value of SEE is equal to 0.195.

In the CoMSIA study, Table 3 indicates that CoMSIA/SEA with the best combination of fields—steric (S), electrostatic (E), and hydrogen bond acceptor (A)—achieved the highest $Q_{cv}^{2}$ value of 0.676. It also achieved an $R_{\text{ncv}}^{2}$ value of 0.805 with two principal components, and a lower SEE of 0.229.

To ensure the robustness of the proposed model, external validation was conducted. As shown in Table 3, the two models, CoMFA and CoMSIA/SEA, have $R_{\text{ext}\;}^{2}$ values of 0.683 and 0.767, respectively, indicating that both models are robust.

### 3.3 Studies of CoMFA and CoMSIA/SEA contour diagram

**FIGURE 2:**
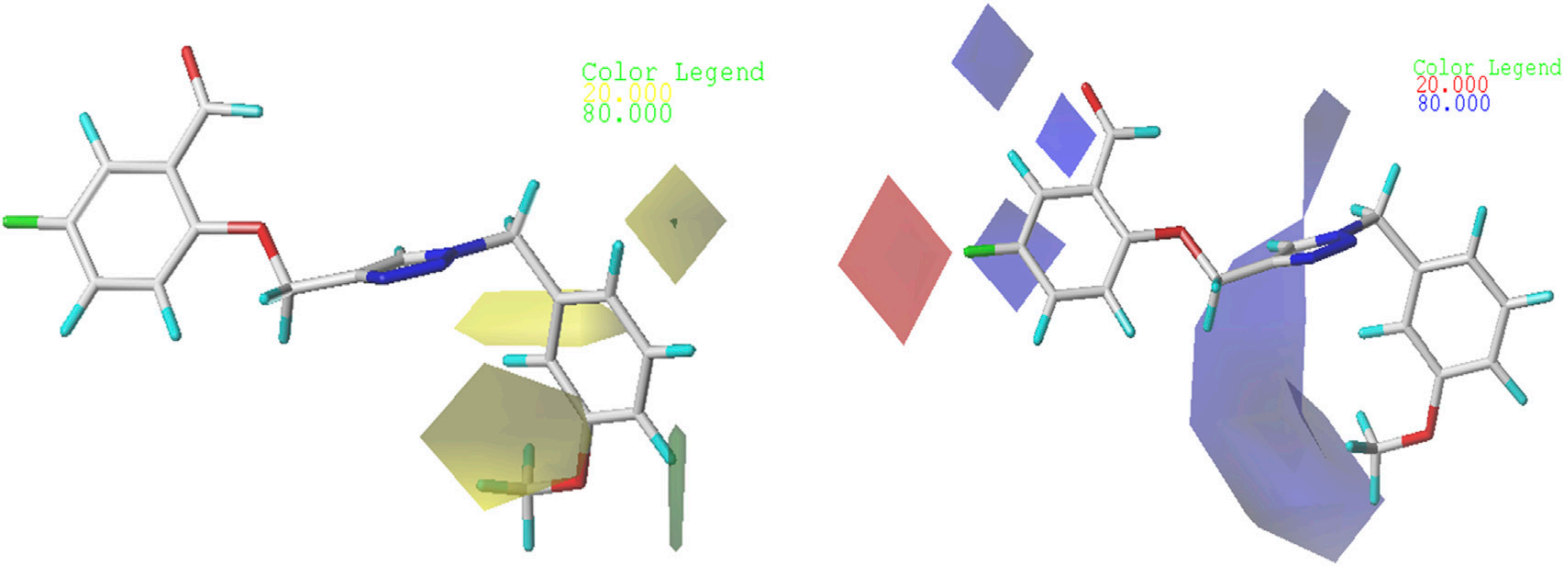
Representation of CoMFA contour maps.

**FIGURE 3:**
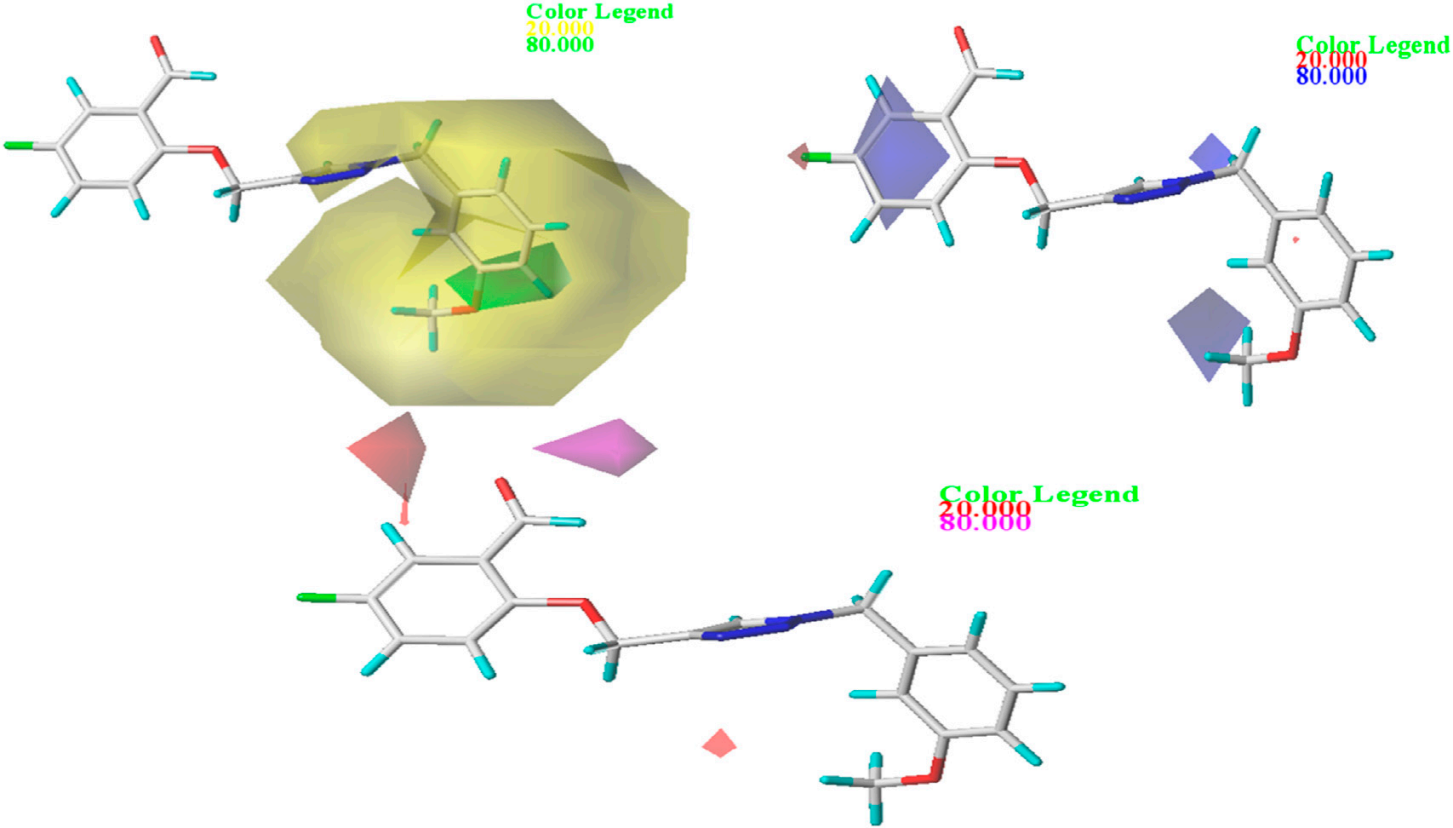
Representation of CoMSIA/SEA contour maps.

In order to gain a deeper comprehension of the investigated molecules’ shape and the different impacts (Steric, electrostatic, etc.) on their biological activity, we employed data visualization techniques to combine elements from the CoMFA and CoMSIA/SEA models. We selected compound N°22, the most active compound, as a reference. The steric, electrostatic, and hydrogen bond acceptor fields of the CoMSIA and CoMFA contour diagrams are shown in Figures 2, 3.

#### 3.3.1 CoMFA contour diagram

So, in the steric contour diagram, the largest contour (green), representing around 80%, indicates regions where bulky groups enhance biological activity. Conversely, the smallest contour (yellow), representing around 20%, marks areas where bulky groups decrease biological activity. For the electrostatic fields, blue contours highlight regions where electron-donating groups boost biological activity, while red contours indicate areas where electron-attracting groups enhance biological activity.

Figure 2 from the CoMFA study display the electrostatic and steric contour diagrams, providing insights into regions that may enhance or diminish the biological activity of derivatives studied. The steric field graph shows a small green area next to the most active molecule, indicating that the steric effect is not favorable to the inhibitory activity. On the other hand, there are three small yellow areas close to the O-CH3 radical, suggesting that this group is not large enough to enhance the inhibitory activity of this compound. Therefore, to enhance the XO inhibitory activity of these compounds, alkyl groups should not be added to the proposed new molecules.

#### 3.3.2 CoMSIA/SEA contour map study

Three distinct fields—stereoscopic, electrostatic, and hydrogen bond acceptor fields—were examined in the context of the CoMSIA model. In Figure 3., the three-contour diagram is displayed.

In Figure 3., the CoMSIA/SEA stereoscopic contour diagram displays a small green contour next to the O-CH3 group and a large yellow contour covering most of the studied molecule, indicating that the molecule is unfavorable to bulky groups. Conversely, the electrostatic contour diagram of the CoMSIA/SEA model shows small blue contours within the studied molecule and an absence of red contours, suggesting that adding electron-withdrawing groups or atoms does not affect the biological activity of the molecule. Instead, introducing electron-donating groups can enhance the inhibitory activity. In the hydrogen bond acceptor domain, magenta and red colors mark favorable and unfavorable positions for hydrogen bond acceptors, respectively. However, in Figure 3., the small magenta and red contours suggest that hydrogen bond acceptors do not significantly influence the biological activity of these derivatives. Therefore, to enhance the OX inhibitory activity of these compounds, it is not necessary to add electron-withdrawing groups in the proposed new molecules.

### 3.4 Molecular interaction analyses

Promising results from molecular anchoring study gave promising results for the molecule synthesis of the two molecules being studied (Molecules N°22 and N°8) for their novel xanthine oxidase inhibitors.

**FIGURE 4:**
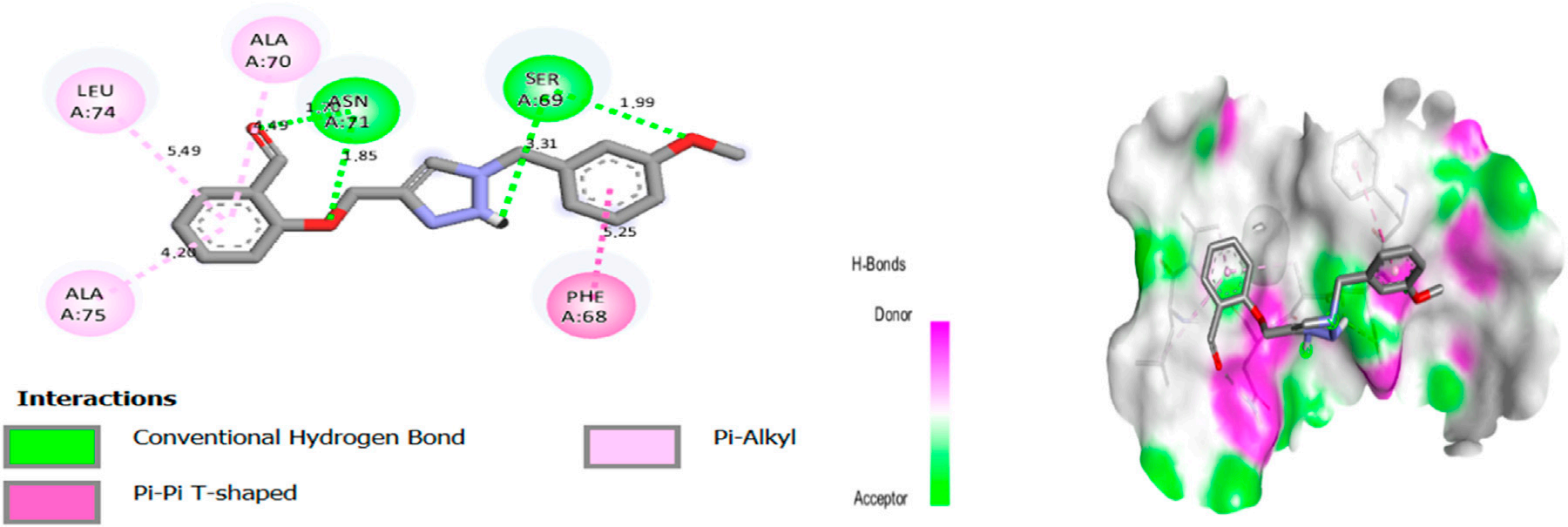
The 2/3D interactions between molecule N°8 with receptor 3NVY.

Molecular docking results for complex N°8-3NVY showed a docking score of −7.22 kcal/mol, suggesting substantial affinity for the targeted enzyme (PDB ID: 3NVY). The results of molecular docking of complex N°8-3NVY are shown in Figure 4.

After viewing the results (Figure 4.), for complex N°8-3NVY, we observe two hydrogen bonds with residues SER 69, and with residues ASN 71, with distances equal to 3.31, and 1.85 Å respectively. There are also three alkyl bonds with residues ALA 70, LEU 74, and ALA 75, with distances equal to 1.49, 5.49, and 4.26 Å respectively. Also, one Pi-Pi T-shaped bond with residue PHE 68, with distances equal to 5.25 Å.

**FIGURE 5:**
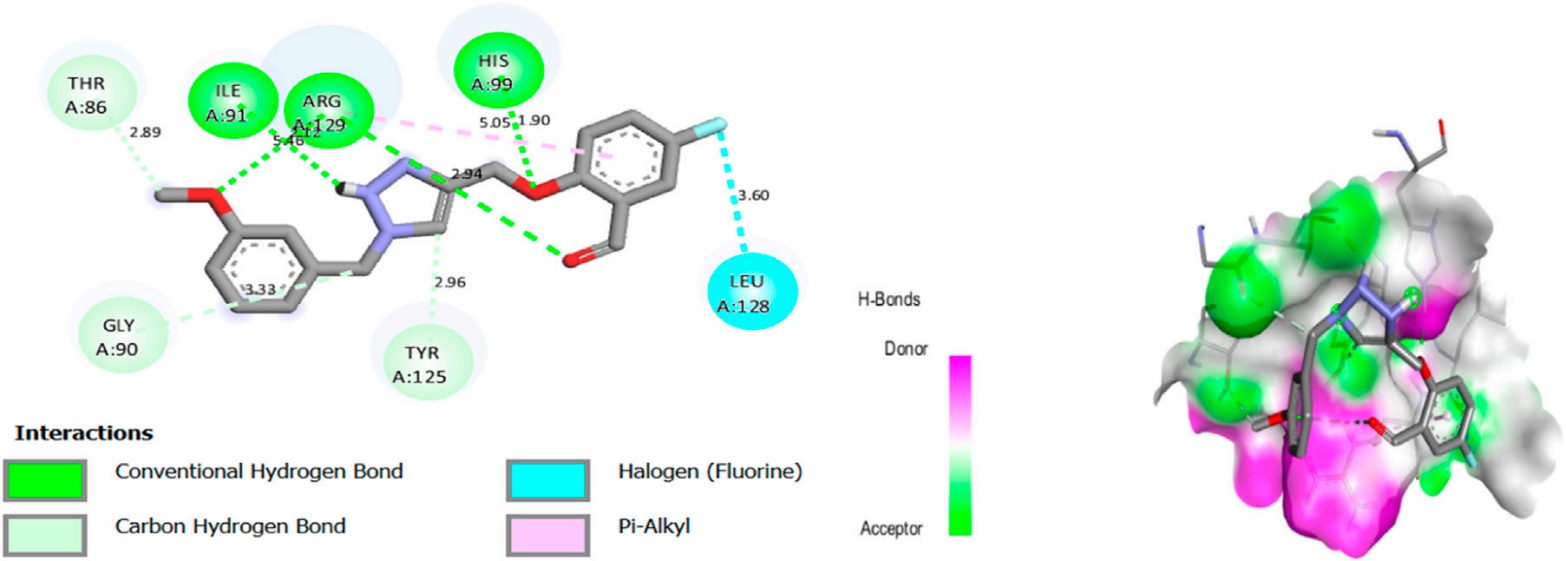
The 2/3D interactions between molecule N°22 with receptor 3NVY.

Molecular docking results for complex N°22 -3NVY showed a docking score of −8.36 kcal/mol, suggesting substantial affinity for the targeted enzyme. The results of molecular docking of complex N°22 -3NVY are shown in Figure 5.

After viewing the results (Figure 5), for complex N°22-3NVY, we observe three hydrogen bonds with residues HIS 99, ARG 29, and with residues ILE 91, with distances equal to 1.90, 2.12 and 5.46 Å respectively. There are also one halogen bonds with residues LEU 128, with distance equal to 3.60 Å. These results suggest that the two components identified can be used as good inhibitors against the receptors studied. These results suggest that synthetic molecules (N°22 and N°8) can be used as inhibitors against the receptors XO.

### 3.5 Molecular dynamics results

An understanding of the stability of the ligand in the protein’s active site and the role important amino acids play in the ligand-protein interaction can be gained through the use of MD modeling (Wang et al., 2018). For molecular dynamics studies up to 100 ns, the two most active molecules (N°8 and N°22) containing the target protein were chosen. The protein interactions with the ligand and the root mean square deviation (RMSD) parameter were used to analyze the MD trajectories.

For researching the stability of ligand docking posture and the role of important amino acids in proteins, MD modeling has shown to be an effective method. In order to evaluate the stability of the complex and observe potential ligand binding modes, we ran 100 ns MD simulations of compounds N°8 and N°22.

**FIGURE 6:**
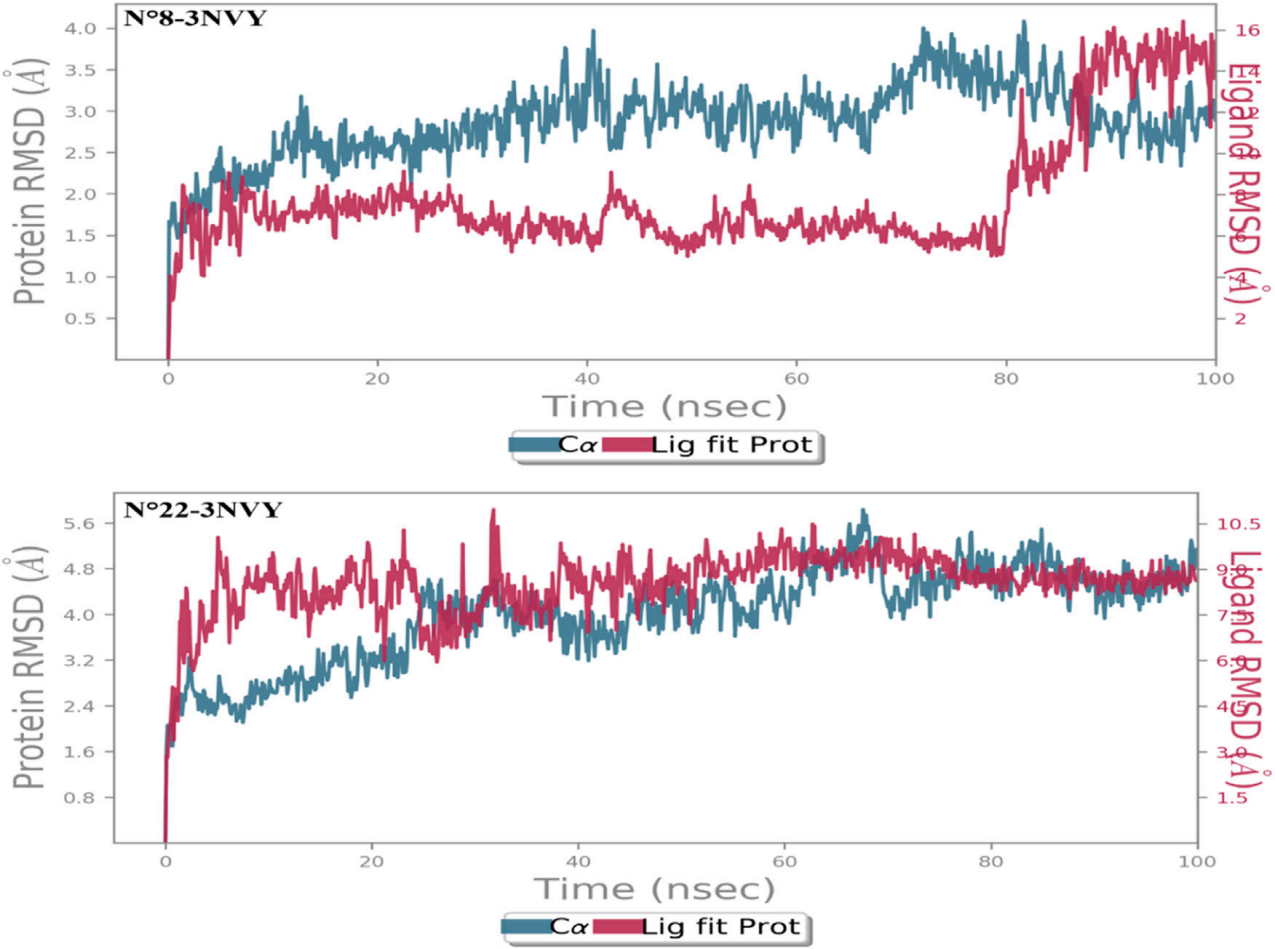
RMSD of two complex N°22-3NVY and N°8-3NVY.

For the first complex N°8-3NVY, as shown in Figure 6, the protein backbone’s RMSD value increased from 1.20 to 4.0 Å during the course of the simulations. The protein configuration’s RMSD value stabilized at about 2.8 Å after 90 ns of simulation. In the same figure, the RMSD of molecule N°8 varies from 4 to 16 Å during the 100 ns. Around 80 ns, there’s a fluctuation down to 8 Å; then there’s another fluctuation around 90 ns down to 4 Å. Thus, the average RMSD between the crystal structure complex and molecule N°8 was wider, indicating that the designed molecule retains no degree of stability within the protein-ligand complex.

For the second complex N°22-3NVY, as shown in Figure 6, the RMSD value of the protein backbone showed a change in RMSD from 2.40 to 5.40 Å throughout the simulation process. Throughout the simulation, the RMSD for the molecule N°22 in Figure 6 fluctuated from 6.50 to 10.50 Å. For the average RMSD of two complex constituents, the average value of the macromolecular structure was around 4.0 Å, while that of molecule N°22 was around 9 Å. Thus, the average RMSD between the crystal structure complex and molecule N°22 was less wide than that of the first complex, indicating that molecule N°22 retains some degree of stability within the protein-ligand complex. Thus, on the basis of the analysis of the two RMSD figures, we conclude that molecule N°22 maintains a certain stability within the protein-ligand complex compared to molecule N°8, which demonstrates lower stability throughout the simulation.

**FIGURE 7:**
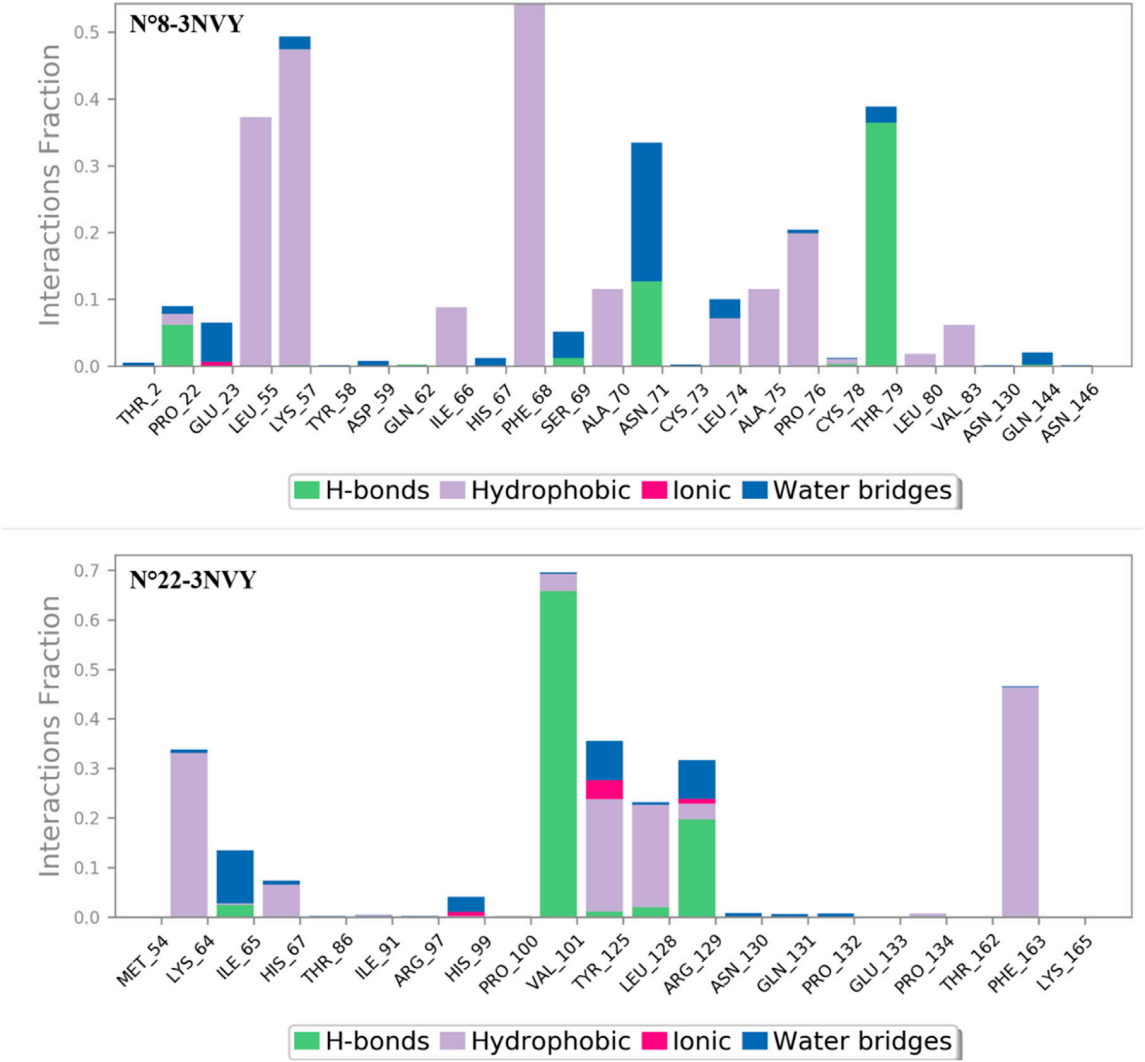
Extensive contacts were found between the macromolecular complexes during the simulation.

The analysis of the contacts observed between the macromolecular complexes bound to the two most active compounds during a 100 ns MD simulation, as shown in Figure 7, revealed the following: the macromolecular structure N°8-3NVY exhibited hydrogen bonding interactions at the amino acid THR 79 (40%) and water bridge bonding 5%). Additionally, the amino acids Phe-68 and LEU 55 showed 60%, 40% hydrophobic interactions respectively, while LYS 57 exhibited 48% hydrophobic interactions and 4% water bridge bonding.

For the second complex N°22-3NVY, the macromolecular structure had the amino acid VAL101 exhibited 65% hydrogen bond interaction, and 5% hydrophobic bond. Also, the amino acid PHE 163 exhibited 50% hydrophobic bonding interaction. Also, the amino acid TYR 125 exhibited 30% hydrophobic bonding interaction and 5% ionic interaction 10% water bridges bonding.

These results indicate that both complexes exhibit strong interactions with various residues of different types, including hydrophobic and hydrogen bond interactions.

**FIGURE 8:**
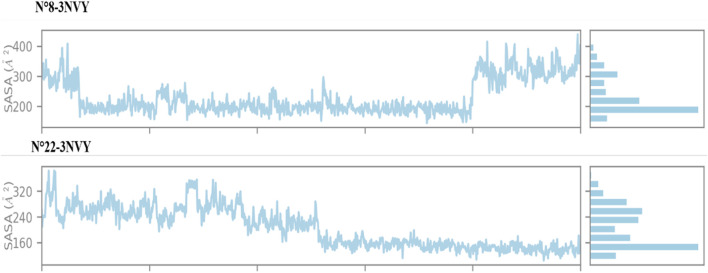
SASA results during the simulation.

The analysis of the solvent-accessible surface area (SASA) for the two most active compounds during a 100 ns MD simulation, as shown in Figure 8, reveals that the SASA for complex N°8-3NVY remains stable, ranging from 200 to 400 Å^2^. In contrast, for complex N°22-3NVY, the SASA varies between 160 and 320 Å^2^. These results suggest good structural complementarity and strong binding between the protein and the ligand in both cases. The stability of the SASA indicates that the ligand is well-positioned within the binding site, with a significant portion of its surface shielded from the aqueous environment. This is generally favorable for efficient and specific interactions with the target protein, as well as for maintaining the stability of the formed complex.

### 3.6 Designing new molecules

**FIGURE 9:**
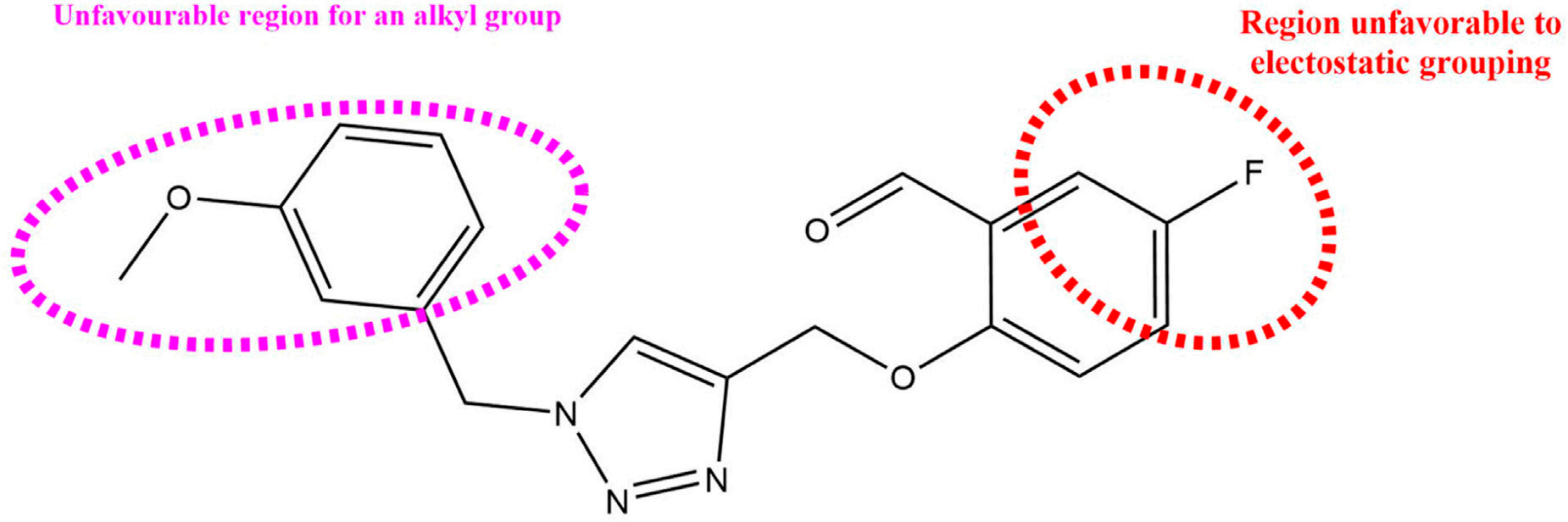
Summary of the structural requirements derived from the 3D-QSAR study analysis.

In order to further our investigation, we employ molecular docking results and CoMFA and CoMSIA contribution diagram analysis to suggest novel compounds that have resemblance to the synthesized ones. Resistance to anti-gout drugs is reaching dangerously high levels worldwide, necessitating the urgent development of new drugs to address this health threat. In response, we have developed new anti-gout drug candidates using guidelines from 3D-QSAR and molecular docking analyses based on the structural properties of the most active molecules. Based on the insights from various methods, including 3D-QSAR analyses contour mapping and molecular docking, we have summarized the structural requirements in Figure 9.

We are proposing new compounds (Table 4), based on the results obtained using a different method and on our own discussions. CoMFA and CoMSIA/SEA were used to predict the activity of the newly added compounds, which were then added to the test set.

**TABLE 4:** Discovery of novel compounds and activity predicted.

ID	Molecules	CoMFA	CoMSIA-SEA
1	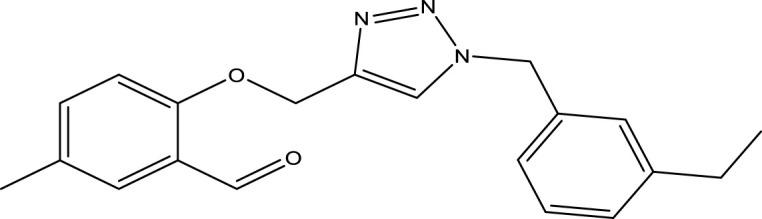	5.7653	5.6494
2	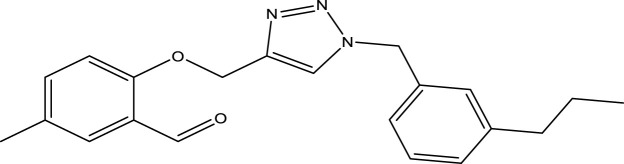	5.767	5.6577
3	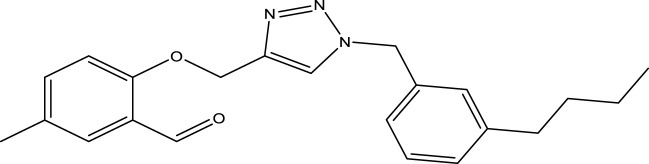	5.7788	5.6622
4	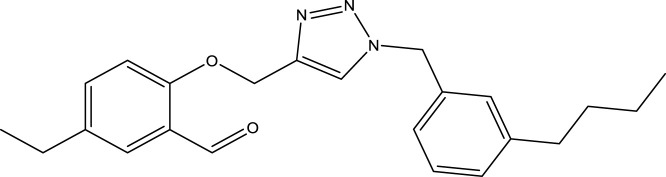	5.7899	5.674
5	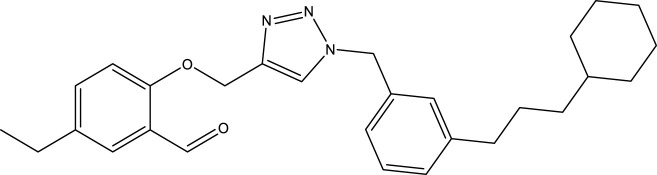	5.7998	5.6846
6	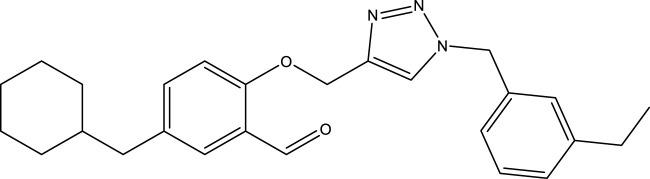	5.7851	5.664

Using the formulas derived from CoMFA and CoMSIA/SEA, we can determine the expected activity of four proposed compounds after changing the groups that affect the inhibitory activity of the suggested molecules. The results for the predicted activities of these 6 new compounds are presented in Table 4. According to Table 4, all 6 new molecules have a predicted activity (pIC_50_) greater than 5.6 for the two proposed QSAR models, and very close to the value of the most active molecule, meaning that the 6 proposed molecules have greater inhibitory activity. Based on the prediction of activities for the new molecules, we are now conducting an analysis of the ADMET properties for the most active new molecules (Pred 4 and Pred 5) and comparing them with the two most active molecules in the studied series (N°22 and N°8).

### 3.7 ADMET proprieties

The primary aim of this study is to predict the medicinal properties of proposed new molecules and then assess their toxicity when used in pharmaceutical applications. The project aims to identify molecules with potential medicinal advantages, while ensuring their safety for therapeutic use.

To enhance the readability of Table 5, we provide brief explanations for the terms LIPO, SIZE, POLAR, INSOLU, INSATU, and FLEX as follows: LIPO denotes lipophilic properties (with a range between −0.7 and 5), SIZE represents molecular weight (ranging from 150 to 500 g/mol), POLAR indicates polarity (measured by topological polar surface area (TPSA) between 20 Å^2^ and 130 Å^2^), INSOLU signifies solubility (with a maximum log S value of 6), INSATU refers to saturation (requiring a fraction of sp^3^ hybridized carbons not less than 0.25), and FLEX denotes flexibility (with no more than 9 rotatable bonds permitted) (Er-Rajy et al., 2024a).

**TABLE 5:** Lipinski’s rule and the oral bioavailability properties of the proposed new molecules and of the most active molecule in the series of molecules studied.

Structure names	Oral bioavailability	Rule of lipinski					
		MW	LogP	N-RB	TPSA	N° H-Ac	N° H-Don
N°8	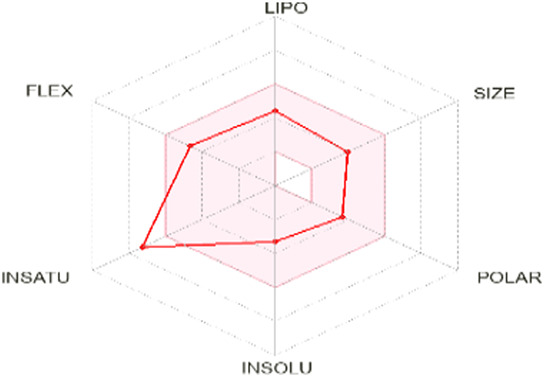	323.35	2.726	7	66.24	6	0
N°22	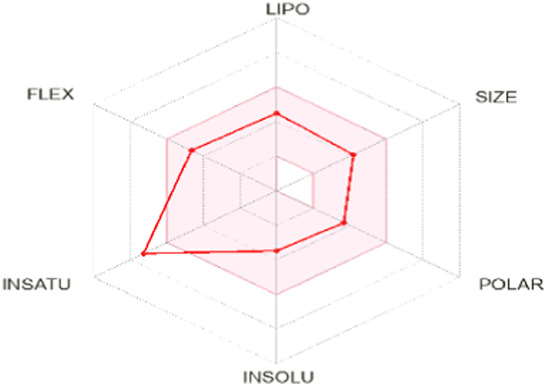	341.34	2.86	7	66.24	6	0
Pred 5	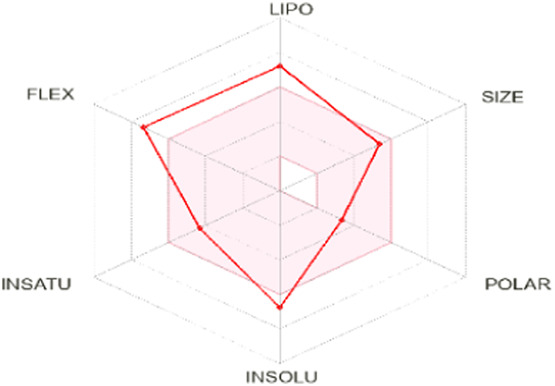	445.60	6.183	11	57.01	5	0
Pred 4	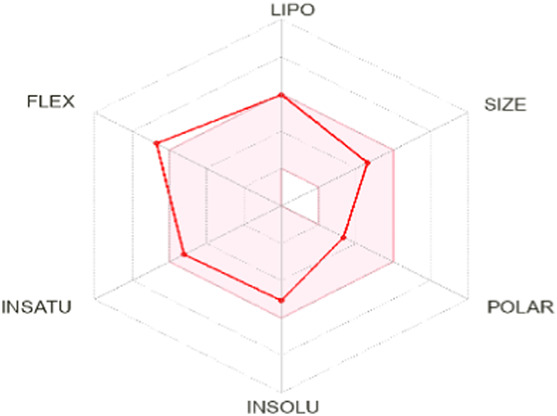	377.48	4.622	10	57.01	5	0

MW: molecular weights, N-RB: number of rotation bonds, N° H-Ac and N° H-Don: number of hydrogens bond acceptors and donors, TPSA: topological polar surface area, Log P: partition coefficient.

Based on oral bioavailability figures, the colored area represents the optimum range for each property. According to the oral bioavailability results for the most active molecules presented in Table 5, such as molecules N°22 and N°8, the oral bioavailability properties are not saturated. However, for our proposed molecule, Pred 5, there is some unsaturation and it falls outside the optimum range in terms of flexibility. Pred 4, on the other hand, is almost within the optimum range for every property.

In order to prevent compounds from entering clinical trials due to toxicity or inadequate pharmacokinetics, they were assessed for similarity to existing drugs and then subjected to ADMET prediction. ADMET prediction results for the proposed new molecules and of the most active molecule in the series of molecules studied are listed in Table 6.

**TABLE 6:** ADMET prediction results of the proposed new molecules and of the most active molecule in the series of molecules studied.

Compounds	Absorptions		Distributions			Metabolisms					Excretion	Toxicity
	Water solubility	Intestinal absorption	VDss	BBB	CNS	Substrate		Inhibitor			Total Clearance	AMES toxicity
						CYP450						
						2D6	3A4	1A2	2C19	2C9		
	Numeric (log mol/L)	Numeric (%absorbed)	Numeric (Log L/kg)	Numeric (Log BB)	Numeric (Log PS)	Categorical (Yes/No)					Numeric (Log mL/min/kg)	Categorical (Yes/No)
N°8	−4.31	98.84	−0.274	−0.205	−2.657	No	Yes	Yes	Yes	Yes	0.174	No
N°22	−4.497	98.31	−0.361	−0.454	−3.305	No	Yes	Yes	Yes	No	0.32	Yes
Pred 4	−6.125	97.81	0.418	−0.251	−2.352	No	Yes	Yes	Yes	Yes	1.648	No
Pred 5	−7.063	96.10	0.465	−0.443	−2.105	No	Yes	No	Yes	Yes	0.861	No

Table 6 shows that the water solubility values are negative, indicating that the proposed new molecules and of the most active molecule in the series of molecules studied is highly soluble in water. Notably, the new molecules we predicted are very soluble in water, with values reaching as low as −7 (Yalkowsky and Valvani, 1980). Also, in terms of intestinal absorption, as shown in Table 6, they showed high intestinal absorption rates in humans, in excess of 98%, suggesting a promising potential for bioavailability.

In terms of distribution, quantified by steady-state volume of distribution (VDss), a log(VDss) value less than −0.15 indicates a relatively low volume of distribution, while a value greater than 0.45 suggests a relatively high volume of distribution (GREENBLATT et al., 1983). Table 6 shows, the VDss values for both molecules N°22 and N°8 are below −0.15, indicating that these two compounds have a relatively low volume of distribution. In contrast, the new molecule Pred 5 has a VDss value greater than 0.45, implying a relatively high volume of distribution for this compound. Similarly, the molecule Pred 4 has a VDss value almost equal to 0.45, indicating that the new molecule Pred 4 also has a relatively high volume of distribution.

The permeation of the blood-brain barrier (BBB) is a crucial property in the pharmaceutical realm as it dictates whether a compound can traverse the BBB, enabling it to exert its therapeutic effects on the brain. The reference standard for BBB permeability classifies it as good if its value is above 0.3, while it is considered poor if it is below −1 (Abraham, 2004). Based on the results obtained, we can conclude that all the studied molecules have average properties for crossing the BBB.

According to the criteria outlined for the central nervous system (CNS) permeability index, compounds with LogPS values above −2 are classified as capable of penetrating the CNS, while those with LogPS values below −3 are considered incapable of penetrating the CNS (Ghose et al., 2012). According to the information provided in Table 6, compound N°22 has a LogPS value below −3, indicating that this compound is unable to penetrate the CNS. However, the new compounds we have proposed have LogPS values between −2 and −3, suggesting that both have the potential to penetrate the CNS.

Cytochrome P450 isoenzymes play a crucial role in drug metabolism within the liver (Zanger and Schwab, 2013). Among these, CYP2D6 and CYP3A4 are particularly notable, as they are responsible for vital detoxification processes in the human body and for modulating drug pharmacokinetics. According to the results obtained, almost all compounds function as inhibitors and substrates of CYP2D6, CYP2C9, CYP3A4 and CYP2C19, and CYP1A2.

The total clearance of a drug provides insights into its half-life, with a low clearance value indicating a longer half-life (Varma et al., 2009). According to the results obtained in Table 6, the four compounds studied have low clearance values, indicating that these compounds have prolonged half-lives for their pharmacological properties.

The Ames toxicity test is a widely used method for assessing toxicity (Xu et al., 2012). From the results obtained, it is evident that the most active molecule (N°22) in the series studied exhibits toxic properties. In contrast, the molecules we have proposed demonstrate non-toxic properties, indicating that they can be used safely.

In conclusion, based on drug similarity studies and ADMET property evaluations, we propose the two molecules we have put forward, Pred 4 and Pred 5, as promising candidates for XO inhibitors. These compounds exhibit favorable absorption, distribution, and metabolism properties, along with low total clearance rates and demonstrated non-toxicity.

### 3.8 DFT analyses

#### 3.8.1 Geometry optimization

**FIGURE 10:**
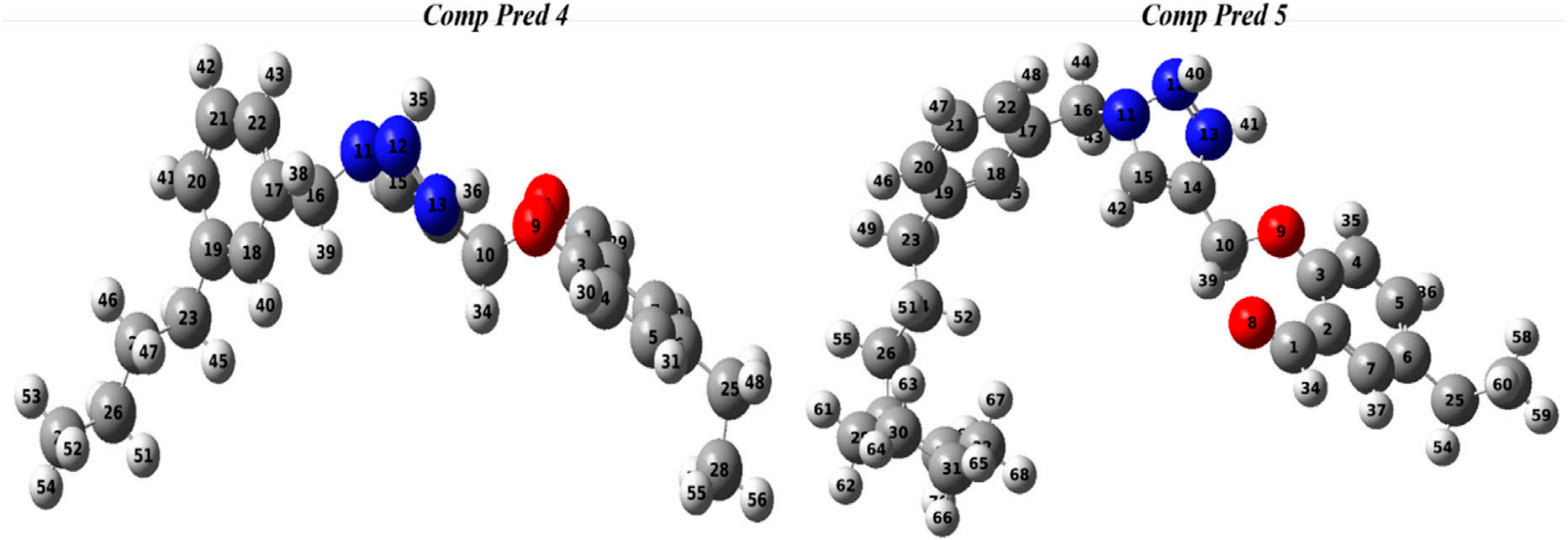
Representation optimal geometries of two predicted molecules (Pred 4, and Pred 5).

Molecular geometry was used to implement a reflected effect on optoelectronic capabilities. A calculation was performed at the DFT method with the 6-311G (d, p) basis set and B3LYP functional to optimize the ground-state configuration of the two new predicted molecules (Pred 4, and Pred 5). Figure 10 shows the optimized geometry of the two new predict molecules with their atom numbers.

#### 3.8.2 Frontier molecular orbitals analysis (FMOs)

FMO analysis is performed to determine the reactivity and nature of the excess electrons in the designed systems (Ahsin et al., 2021). The Occupied Molecular Orbital (HOMO) energy dictates the molecule’s capacity to donate an electron, on the other hand, the Lowest Unoccupied Molecular Orbital (LUMO) energy dictates its ability to accept one (Er-Rajy et al., 2022b). The disparity in energy between the HOMO and LUMO is pivotal in shaping the molecule’s chemical reactivity. A narrow energy differential (HOMO-LUMO) suggests a diminished in kinetic stability, as it is energetically more favorable to add electrons to a low LUMO and receive electrons from a high HOMO. Furthermore, calculated energy values and global descriptors are presented in Table 7.

**TABLE 7:** Global reactivity descriptors were computed for the two predicted molecules (Pred 4, and Pred 5).

Parameters	Pred 4	Pred 5
E_LUMO_/eV	−2.033	−2.035
E_HOMO_/eV	−5.146	−5.151
E_gap_/eV	3.113	3.115
Ionization energy [I = -E_HOMO_]/eV	5.146	2.035
Electron Affinity [A = -E_LUMO_]/eV	2.033	5.151
Chemical Hardness η=(I-A)/2]/eV	1.556	1.557
Chemical Potential [u = -(I + A)/2]/eV	−3.590	−3.593
Softness of Molecule (s = I/2 η]/eV-1	1.653	0.653
Electronegativity [x = I + A)/2]/eV	3.590	3.593
Electrophilicity Index (ω = u^2^/2η]/eV	4.139	4.143

According to Table 7, for the new molecules predicts, the HOMO and LUMO values of Pred 4 compound have been calculated to be −5.145 eV and −2.033 eV respectively. This means that the energy difference between HOMO and LUMO has been calculated to be 3.112 eV, indicating a difference that is not significant. This energy difference suggests a charge transfer interaction within the compounds studied. Based on these results, Pred 4 exhibits an average energy gap ( $E_{\text{gap}}$ = 3.112 eV), indicating a moderate level of polarizability and reactivity.

For the Pred 5 compound, the HOMO and LUMO values have been calculated to be −5.151 eV and −2.035 eV respectively. This means that the energy difference between HOMO and LUMO has been calculated to be 3.115 eV. This energy difference suggests an average charge transfer interaction within the compounds studied. Based on these results, Pred 4 has a medium energy gap, making it more polarizable and therefore more reactive than molecule Pred 5.

**FIGURE 11:**
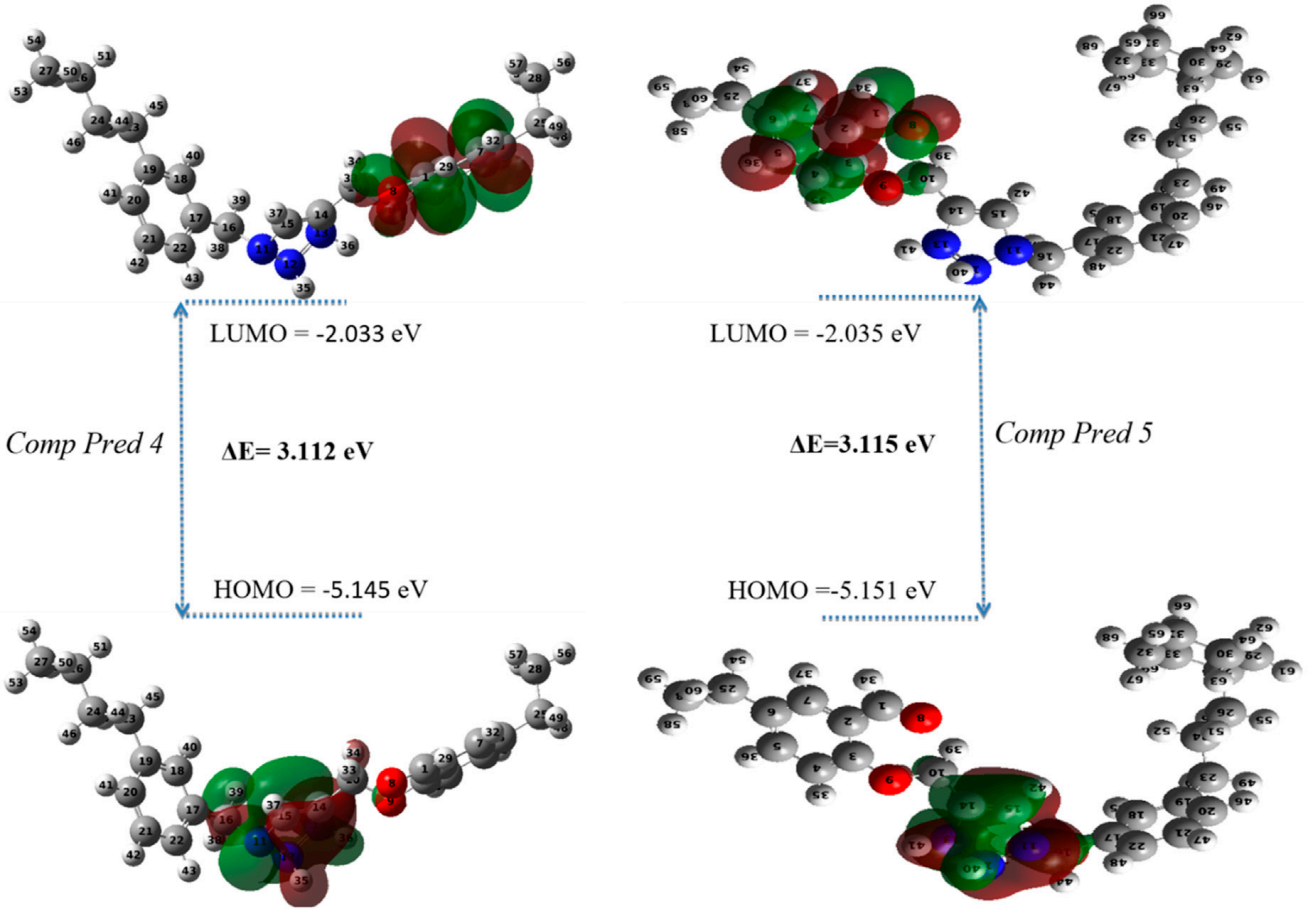
The FMOs of two predicted molecules (Pred 4, and Pred 5).

To better understand where the electron density is located for LUMO and HOMO, we display the electron density of each level in Figure 11. Figure 11 displays 3D plots or distributions of the HOMO and LUMO for two predicted molecules (Pred 4, and Pred 5).

According Figure 11, the new Pred 4 molecule, the HOMO orbital is located on the triazole fragment. But the LUMO orbital is located on the 2-methoxybenzaldehyde group. In other words, the two groups triazole and 2-methoxybenzaldehyde are electron donor and acceptor regions respectively. The other new molecule, Pred 5, gives the same results as Pred 4, so the interpretation will be the same. So, the two new predicted molecules are therefore reactive.

#### 3.8.3 Molecular electrostatic potential analysis (MEP)

**FIGURE 12:**
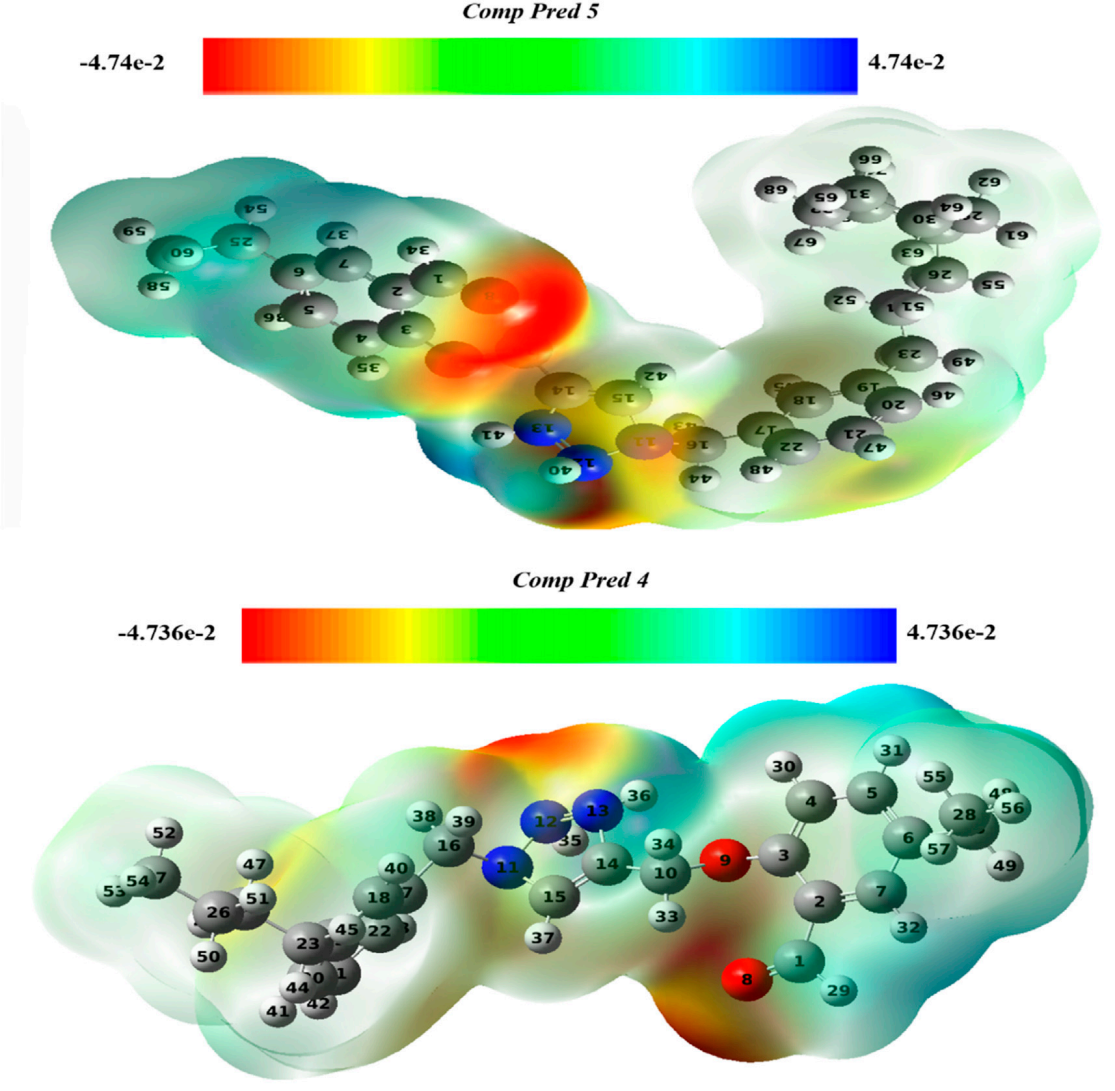
The MEP maps for two predicted molecules (Pred 4, and Pred 5).

By examining the charge distribution and photophysical properties, an electrostatic potential map can be utilized to deduce the reactivity of chemical substances. It helps to pinpoint regions vulnerable to nucleophilic and electrophilic attacks (Asif et al., 2023). The system under investigation was simulated using DFT method with the 6-311G (d, p) basis set and B3LYP functional, to analyze its electrophilic and nucleophilic regions. Figure 12 depicts the MEP charts for two predicted molecules (Pred 4, and Pred 5), along with their respective indicators.

Graphically, the electron density and charge distribution of each molecule are illustrated by distinctive colored bands. In the MEP map, blue indicates electron-rich sites with surplus electrons available for electrophilic attack, while red represents electron-poor sites sensitive to nucleophilic attack (Costa et al., 2017). Additionally, green is utilized to denote neutral sites within the molecules.

According to the results obtained, the color scale for the two new Pred 4 and Pred 5 molecules ranges from −0.74 e-2 to 0.74 e-2, as shown in Figure 12. In the figures of the two predicted new molecules, we notice that there is only one region colored in red in the same position for both molecules, next to the 2-methoxybenzaldehyde, particularly next to the oxygen atom. This indicates that the 2-methoxybenzaldehyde group is electron-poor and thus accessible for an attack by a nucleophilic.

#### 3.8.4 The non-covalent interaction analysis (NCI)

Using NCI diagrams generated by the Multiwfn program, we probed the degree of engagement between dopant and surface in intermolecular interactions (Lu and Chen, 2012). These diagrams offer valuable insights into the interactions between atoms in doped (For two predicted molecules (Pred 4, and Pred 5)), often referred to as weak forces. Also, by RDG approach is employed to identify both intermolecular and intramolecular non-covalent. It is a visualization index based on electron density and its derivatives, utilizing RDG results at low density regions (Amraoui et al., 2024). These interactions encompass steric hindrance, van der Waals forces, hydrogen bonding and various others.

**FIGURE 13:**
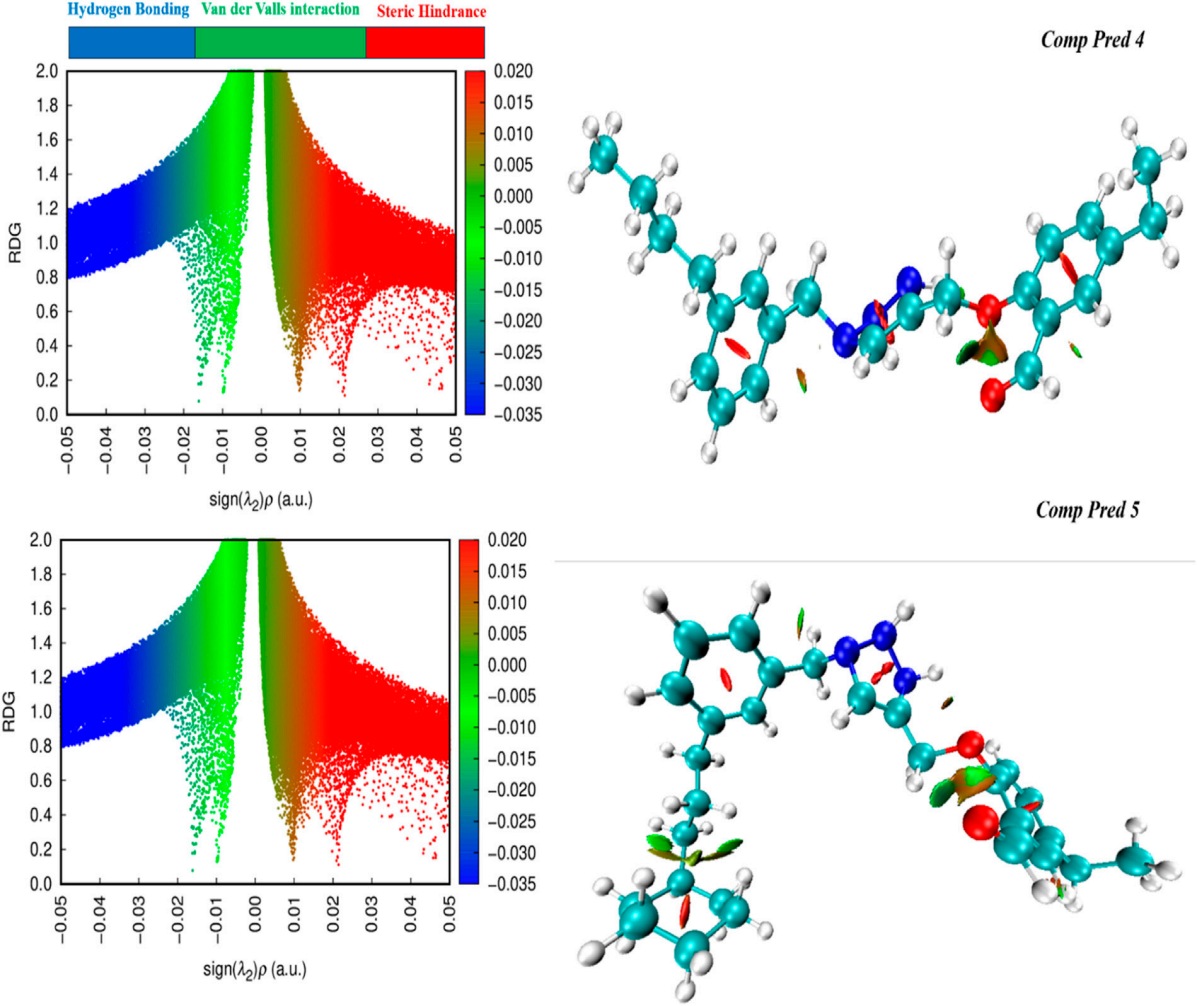
RDG-NCI pictures for two predicted molecules (Pred 4, and Pred 5).

Therefore, the study conducted by the NCI predicted the consistency of the system. The NCI scatter plot shows two functions, namely the electron density sign λ_2_(ρ) for the x-axis and the RDG for the y-axis. This plot shows the cross-section and the type of physical forces involved in the interactions. A colored three-dimensional iso-surface of two Pred 4, and Pred 5 molecules highlights the attraction of intramolecular interaction, as shown in Figure 13(left). Strong attractive forces are indicated by the sign λ_2_(ρ) less than zero, and the area of the graph where λ_2_(ρ) is greater than zero represents areas of repulsive interactions. In addition, points with sign λ_2_(ρ) equal to zero that describe interactions due to van der Waals forces are placed here (Ali et al., 2024).

As a result, the electron density and RDG functions give an accurate NCI mapping of the two molecules predicted (Pred 4 and Pred 5). The NCI graphs of two molecules predicted depicted in Figure 13 (right) illustrate various interactions:

✓ Weak contacts, identified as van der Waals forces, are shown in green and identified between the oxygen atoms of the 2-methoxybenzaldehyde group for the two predicted molecules (Pred 4 and Pred 5).
✓ Attractive forces, known as hydrogen bonds, are indicated by the red ring, which exists at the center of both benzene rings, as well as at the center of the triazole group for the two predicted molecules (Pred 4 and Pred 5).

So, the two molecules we have proposed possess significant properties such as stability and reactivity levels; they also exhibit weak intramolecular interactions.

## 4 Conclusion

A xanthine oxidase inhibitor reduces uric acid production by inhibiting the enzyme xanthine oxidase, making it useful for treating hyperuricemia and related conditions such as gout. A series of 4-(phenoxymethyl)-1H-1,2,3-triazole with anti-gout activity have been studied computationally. By computational study of this series, we built two QSAR models. The two CoMFA, CoMSIA/SE models showed good reliability and predictability. Both models were successfully evaluated using external and internal validation methods. Analysis of the contour maps of the different models provides structural information for optimizing the inhibitory activity of the inhibitors studied. In addition, the molecular docking study shows that the two selected compounds (N°8 and N°22) and the target protein (PDB ID: 3NVY) have established significant interactions with very important amino acids. A molecular dynamics study revealed that the N°22-3NVY complex remained highly stable throughout the simulation. These results were used to develop new compounds and predict their inhibitory activities. Next, physicochemical and ADMET analyses showed that the two predicted molecules (Pred 4 and Pred 5) have drug-like properties. Next, we used DFT study to evaluate the chemical reactivity properties of the two most active proposed compounds (Pred 4 and Pred 5). The results of the DFT study revealed that both molecules exhibit moderate chemical stability and reactivity. Therefore, these two proposed compounds can be used as inhibitors of xanthine oxidase, thereby reducing the production of uric acid by inhibiting this enzyme. This makes them candidates for treating hyperuricemia and related conditions, such as gout.

## Data Availability

The original contributions presented in the study are included in the article/supplementary material, further inquiries can be directed to the corresponding authors.
